# Supplementary material for: Low Doses of PFOA Promote Prostate and Breast Cancer Cells Growth through Different Pathways
Source: Int J Mol Sci. 2022 Jul 18;23(14):7900. doi: 10.3390/ijms23147900 (PMC9318902; doi:10.3390/ijms23147900)
Supplement: Supplementary file 1 [file ijms-23-07900-s001.zip › ijms-1817936-supplementary.pdf]

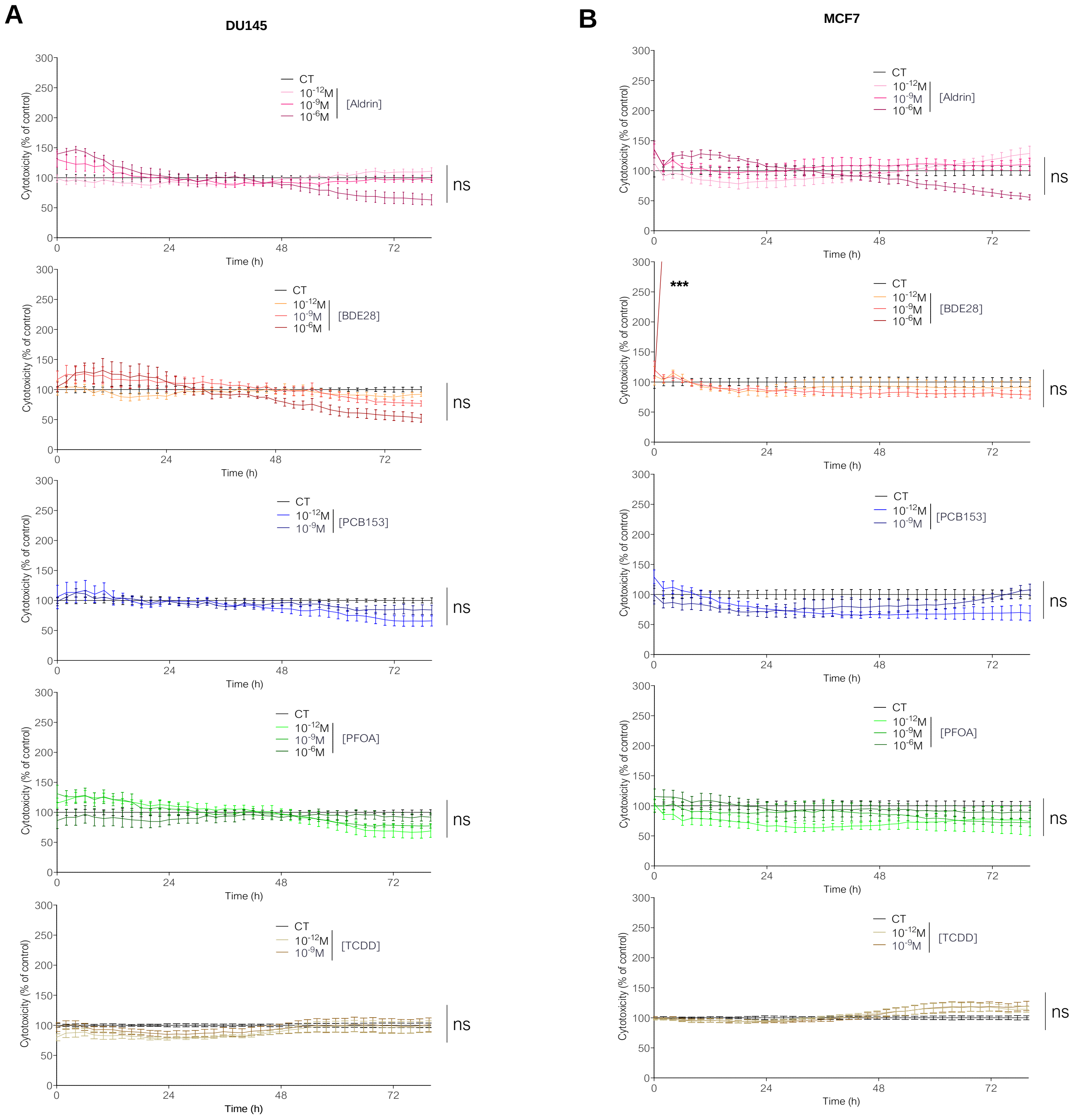

Figure S1: IncuCyte® cytotoxicity assay of **(A)** DU145 and **(B)** MCF7 after treatment of 80h with either Aldrin (10^{-12}M, 10^{-9}M or 10^{-6}M), BDE28(10^{-12}M, 10^{-9}M or 10^{-6}M), PCB153(10^{-12}M or 10^{-9}M), PFOA(10^{-12}M, 10^{-9}M or 10^{-6}M) or TCDD (10^{-12}M or 10-9M) in comparison with cells treated with EtOH (CT). Each point represent a mean ± SEM of triplicates. Three wells were used per replicates and 9 pictures were taken per well. One way anova compared to EtOH treated cells.

*p<0,05; **p<0,005; ***p<0,0005

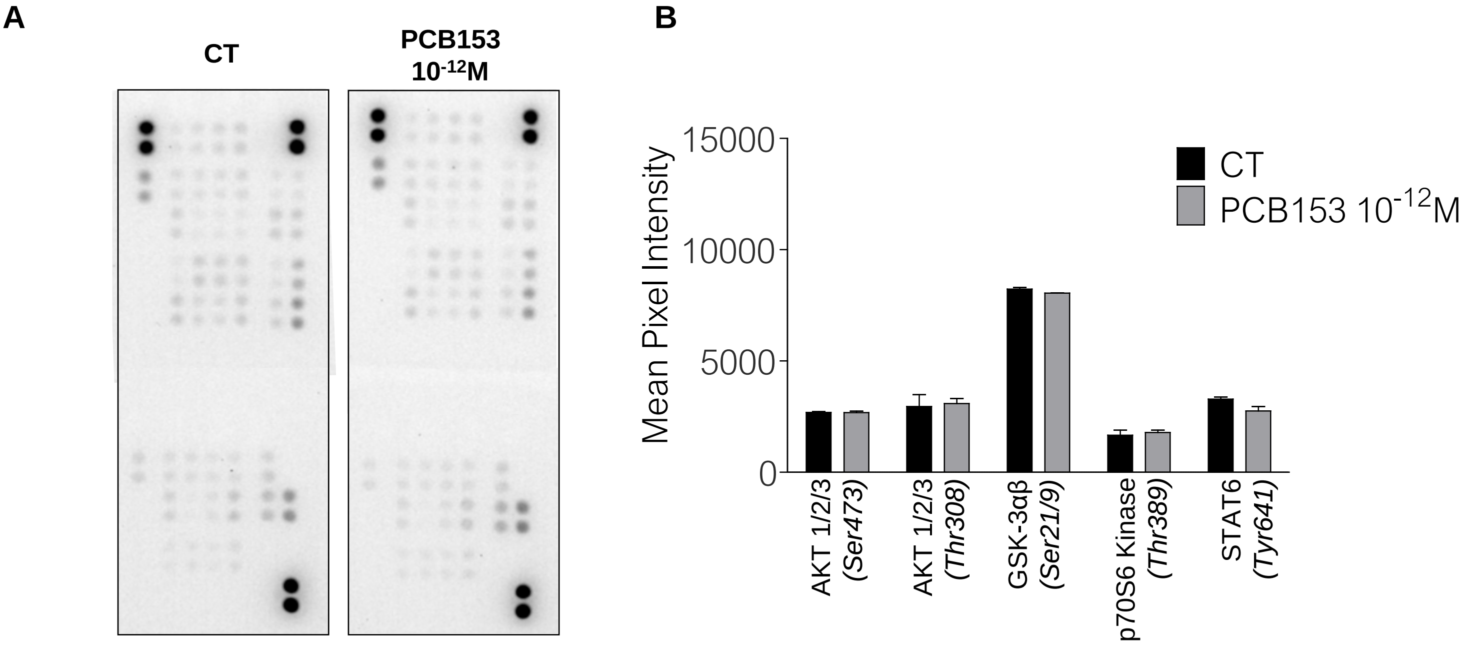

Figure S2 Human Phospho-Kinase Array. Parallel determination of the relative levels of protein phosphorylation of a range of 43 kinase phosphorylation sites of DU145 after 48h of treatment of PCB153 (10^{-12}M) in comparison with cells exposed with EtOH (CT) : **(A)** image and **(B)** quantification (no statistic test : two points each).

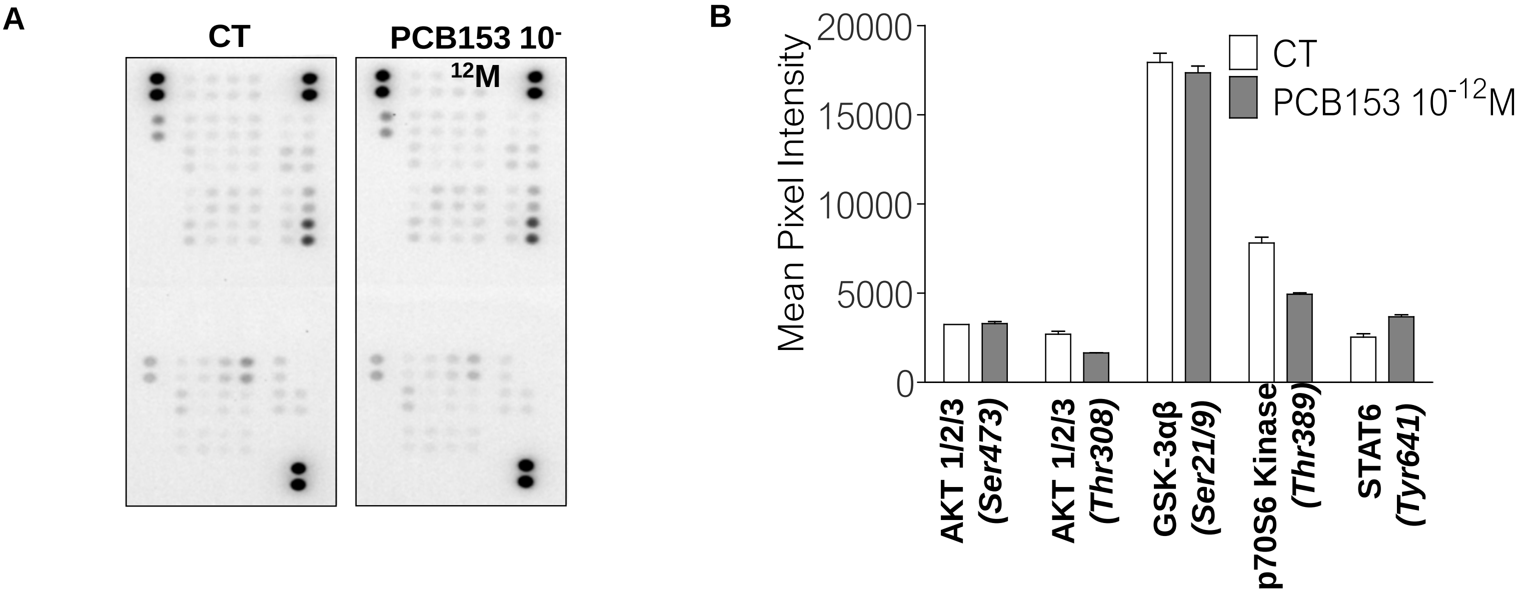

Figure S3 Human Phospho-Kinase Array. Parallel determination of the relative levels of protein phosphorylation of a range of 43 kinase phosphorylation sites of MCF7 after 48h of treatment of PCB153 (10^{-12}M) in comparison with cells exposed with EtOH (CT) : **(A)** image and **(B)** quantification (no statistic test : two points each).

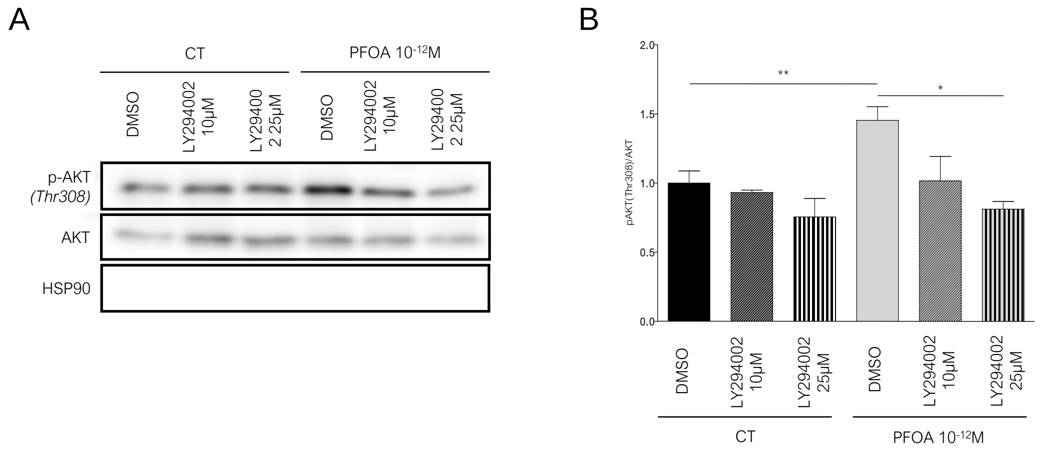

Figure S4: Western blot of phospho-AKT(Thr308), total AKT and HSP90 of MCF7 protein extract after 48h of treatment with PFOA (10^{-12}M) and either DMSO, LY294002 (10µM or 25µM) overnight exposition. B) Quantification of protein level of three different western blot experiments. T test. *p<0,05; **p<0,005; ***p<0,0005

Protein	Source	Identifier
pAKT (Ser473)	Cell Signaling	4058
pAKT (Thr308)	Cell Signaling	4056
AKT total	Cell Signaling	4685
phopsho-GSK3αβ (Ser21/9),	Cell Signaling	8566
GSK3αβ total	Cell Signaling	5676
phospho-p70S6Kinase (Thr389)	Cell Signaling	9234
p70S6Kinase total	Cell Signaling	2708
HSP90	Santa Cruz	sc-13119
Actine	Fisher Scientific	MA5-15739

Table S1 : List of antibodies used for Western Blot

Gene	Sequence (5' -> 3')	
PlexinD1	F	AATGGGCGGAACATCGTCAAG
	R	CGAGACTGGTTGGAAACACAG
RPLP0	F	AGGTGTAATCCGTCTCCACAGA
	R	GCATCAGTACCCAATTCTATCAT

Table S2 : Primers used for RT-qPCR.
